# CEACAM1 long isoform has opposite effects on the growth of human mastocytosis and medullary thyroid carcinoma cells

**DOI:** 10.1002/cam4.1050

**Published:** 2017-03-23

**Authors:** Chiyuki Ueshima, Tatsuki R. Kataoka, Yusuke Takei, Masahiro Hirata, Akihiko Sugimoto, Mitsuyoshi Hirokawa, Yoshimichi Okayama, Richard S. Blumberg, Hironori Haga

**Affiliations:** ^1^ Department of Diagnostic PathologyKyoto UniversityKyotoJapan; ^2^ Department of Diagnostic PathologyKuma HospitalHyogoJapan; ^3^ Division of Molecular Cell Immunology and AllergologyAdvanced Medical Research CenterNihon University Graduate School of Medical ScienceTokyoJapan; ^4^ Gastroenterology DivisionDepartment of MedicineBrigham and Women's HospitalHarvard Medical SchoolBostonMassachusetts

**Keywords:** CEACAM1, mast cell, medullary thyroid carcinoma, SHP‐1, Src family kinases

## Abstract

Carcinoembryonic antigen‐related cell adhesion molecule 1 (CEACAM1) is expressed in a number of tumor cell types. The immunoreceptor tyrosine‐based inhibitory motif (ITIM)‐containing isoforms of this molecule which possess a long cytoplasmic tail (CEACAM1‐L) generally play inhibitory roles in cell function by interacting with Src homology 2 domain‐containing tyrosine phosphatase (SHP)‐1 and/or SHP‐2. Src family kinases (SFKs) are also known to bind to and phosphorylate CEACAM1‐L isoforms. Here, we report that CEACAM1 was uniquely expressed at high levels in both human neoplastic mast cells (mastocytosis) and medullary thyroid carcinoma cell (MTC) lines, when compared with their expression in nonneoplastic mast cells or nonneoplastic C cells. This expression was mainly derived from CEACAM1‐L isoforms based upon assessment of CEACAM1 mRNA expression. CEACAM1 knockdown upregulated cell growth of HMC1.2 cells harboring KIT mutations detected in clinical mastocytosis, whereas downregulated the growth of TT cells harboring RET mutations detected in clinical MTCs. Immunoblotting, ELISA and immunoprecipitaion analysis showed that activated SHP‐1 is preferentially associated with CEACAM1 in HMC1.2 cells harboring KIT mutations, whereas Src family kinases (SFKs) are preferentially associated with CEACAM1 in TT cells harboring RET mutations. These studies suggest that the dominantly interacting proteins SHP1 or SFK determine whether CEACAM1‐L displays a positive or negative role in tumor cells.

## Introduction

The carcinoembryonic antigen‐related cell adhesion molecule (CEACAM) family comprises a group of heavily glycosylated molecules characterized by extracellular domains with immunoglobulin‐related structures 1. Carcinoembryonic antigen‐related cell adhesion molecule 1 (CEACAM1), also known as CD66a or biliary glycoprotein‐1, is commonly expressed in various tumor cell types 1. A number of splice variants of CEACAM1 are described in the human 2. These variants differ with respect to the number of extracellular domains or type of intracellular cytoplasmic domains. In the case of the extracellular domains, they consist of one amino‐terminal immunoglobulin variable‐region‐like (IgV‐like) domain and a maximum of three immunoglobulin constant‐region‐type‐2‐like (IgC2‐like) domains. In the case of the cytoplasmic domains, these various isoforms are connected via splicing to either a long cytoplasmic tail (L) containing two immunoreceptor tyrosine‐based inhibitory motifs (ITIMs) or a short cytoplasmic tail (S) that lacks ITIMs. The IgV‐like domains mediate hemophilic or heterophilic interactions 3, 4, whereas the roles of the varying number of IgC2‐like domains remain unclear. The intracellular ITIMs coordinate inhibitory signaling by recruiting Src homology 2 domain‐containing tyrosine phosphatase (SHP)‐1 or SHP‐2 following phosphorylation by Src family tyrosine kinases 5. SHP‐1 and SHP‐2 are nonreceptor tyrosine phosphatases, which inhibit signaling by reversing critical tyrosine phosphorylation reactions induced by the action of tyrosine kinases 6. Thus, the ITIM‐containing family members of CEACAM1 (CEACAM1‐L) mediate negative signals, whereas ITIM‐deficient CEACAM1 (CEACAM1‐S) isoforms do not 1. An increased ratio of CEACAM1‐L / CEACAM1‐S has been reported to be associated with decreased proliferation of tumor cells 7. In addition to SHP‐1 and SHP‐2, the ITIM of CEACAM1 can also bind Src family kinases (SFKs), which play critical signaling roles in hematopoietic cell function, including activation of B cells, T cells, NK cells, monocytes, granulocytes, and mast cells 8. SFKs binding to CEACAM1 are thought to contribute to cell adhesion properties of eosinophils as well as tumors 9, 10, 11. SFK phosphorylation of CEACAM1 allows for CEACAM1 binding to SHP1 or SHP2 thus promoting inhibitory ITIM function 12. We previously observed that CEACAM1 is expressed in the LAD3 human neoplastic mast cell line 13. However, the role of CEACAM1 in the functions of mast cells or mast cell lines remains unknown.

Mast cells are cells of hematopoietic origin which, in addition to participating in innate and acquired immune responses, are central for the initiation of allergic reaction 14. The growth factor receptor with inherent tyrosine kinase activity, KIT, is essential for mast cell growth, differentiation and survival 15, and gain of function mutations in KIT allow the dysregulated growth of mast cells associated with the myeloproliferative disorder, mastocytosis 16, 17. Multiple ITIM‐bearing receptors are expressed on mast cells, and we and others have demonstrated that such receptors have the capacity to inhibit the growth of these cells 18, 19, 20, 21, 22, 23, 24. Generally, these receptors mediate inhibitory signals through interactions with SHP‐1, SHP‐2, or Src homology 2 domain‐containing inositol 5‐phosphatase 1 5, resulting in the suppression of normal or mutated KIT signals through the respective downregulation of tyrosine kinase or phosphatidylinositol 3‐kinase‐mediated responses 18, 19, 20, 21, 22, 23, 24.

RET is another growth factor receptor, and gain‐of‐function type mutations in this receptor drives neoplastic change in C cells in the thyroid gland (medullary thyroid carcinoma [MTC]) 25. Two of four cases of clinical MTCs were reported to be CEACAM1‐positive 26. The role of CEACAM1 in the functions of MTCs remains unsettled. We therefore examined the expression and function of CEACAM1 in neoplastic mast cells (mastocytosis) and MTCs of human origin.

## Materials and Methods

### Donors

At Kyoto University Hospital (Sakyo‐ku, Kyoto, Japan); skin biopsies were performed on 19 mastocytosis patients and 4 nonmastocytosis patients (chronic dermatitis, nonspecific). These patients signed the “Kyoto University Hospital Informed Consent Form for the Non‐therapeutic Use of Histopathological Materials”, and the signed forms have been uploaded into all electronic health records. All mastocytosis patients were diagnosed according to the World Health Organization criteria. The clinical characteristics of these patients are summarized in Table 1.

**Table 1:** Clinical characteristics of skin biopsy samples donors with mastocytosis

Case	Age (years)	Gender	diagnosis	CEACAM1 expression
1	0	F	Systemic mastocytosis	Negative
2	0	M	Cutaneous mastocytosis	Negative
3	0	F	Cutaneous mastocytosis	Positive
4	0	F	Cutaneous mastocytosis	Positive
5	0	M	Cutaneous mastocytosis	Negative
6	1	M	Cutaneous mastocytosis	Negative
7	1	F	Cutaneous mastocytosis	Negative
8	2	M	Cutaneous mastocytosis	Negative
9	21	M	Cutaneous mastocytosis	Negative
10	29	M	Cutaneous mastocytosis	Negative
11	30	F	Cutaneous mastocytosis	Negative
12	39	M	Cutaneous mastocytosis	Negative
13	42	M	Cutaneous mastocytosis	Negative
14	47	F	Cutaneous mastocytosis	Negative
15	51	M	Systemic mastocytosis	Negative
16	51	F	Cutaneous mastocytosis	Positive
17	59	M	Cutaneous mastocytosis	Positive
18	63	M	Systemic mastocytosis	Positive
19	64	M	Cutaneous mastocytosis	Negative

All cases were immunohistochemically tryptase‐positive.

Medullary thyroid carcinomas were diagnosed and surgically resected in Kuma Hospital (Kobe, Hyogo, Japan) after written permission was obtained from each patient. The clinical characteristics of the patients are summarized in Table 2.

**Table 2:** Clinical characteristics of surgical samples donors with medullary thyroid carcinoma in this study

Case	Age (years)	Gender	RET mutation	CEACAM1 expression
1	60	F	Negative	Positive
2	65	M	Negative	Positive
3	51	F	Negative	Positive
4	66	F	Negative	Positive
5	32	F	Negative	Negative
6	64	F	Negative	Positive
7	54	F	Positive (MEN)	Positive
8	36	F	Positive (MEN)	Positive
9	26	F	Positive (MEN)	Positive
10	26	F	Negative	Positive
11	61	M	Negative	Positive
12	44	F	Positive (sporadic)	Positive
13	42	F	Positive (MEN)	Positive
14	66	M	Negative	Negative
15	64	F	Negative	Negative
16	38	F	Negative	Positive
17	9	F	Positive (sporadic)	Positive
18	54	F	Negative	Negative
19	36	M	Negative	Negative
20	61	F	Positive (sporadic)	Positive
21	77	F	Negative	Negative
22	80	M	Negative	Positive

All cases were immunohistochemically calcitonin‐positive.

MEN; multiple endocrine neoplasia syndrome.

### Cells

Human mast cells (HuMCs) were obtained from peripheral blood progenitor cells using the lineage‐negative cells (CD4^−^ / CD8^−^ / CD11b^−^ / CD14^−^ / CD16^−^ / CD19^−^) which were obtained using MicroBeads (Miltenyi Biotec., Auburn, CA). The cells were cultured in METHOCULT SFBIT H4236, containing 1.2% methylcellulose with rhSCF (200 ng/mL, Peprotech, Rocky Hill, NJ), rhIL‐6 (50 ng/mL, Peprotech), and rhIL‐3 (5 ng/mL, Peprotech) 27.

The human LAD2 mastocytosis cell line was cultured in StemPro‐34 medium, with supplement, containing _L_‐glutamine (2 mmol/L), penicillin (100 units/mL), streptomycin (100 *μ*g/mL), and rhSCF (100 ng/mL, Peprotech) 28.

The HMC1.1 (expresses V560G KIT mutation) and HMC1.2 (expresses V560G and D816V KIT mutations) human mastocytosis cell lines were grown in IMDM medium supplemented with FBS (10%), _L_‐glutamine (2 mmol/L), penicillin (100 units/mL), and streptomycin (100 *μ*g/mL) 17, 29, 30.

The K562, the 293T, the HT‐29 and the Jurkat cell lines were purchased from American Type Culture Collection (ATCC, Manassas, VA), and grown in RPMI1640 medium supplemented with FBS (10%), _L_‐glutamine (2 mmol/L), penicillin (100 units/mL), and streptomycin (100 *μ*g/mL). The TT cell lines were also purchased from ATCC, and grown in F‐12 medium supplemented with FBS (10%), _L_‐glutamine (2 mmol/L), penicillin (100 units/mL), and streptomycin (100 *μ*g/mL).

### Antibodies, cytokines, and reagents

The mouse anti‐human CEACAM1 monoclonal antibody (Ab) GM8G5 (mouse monoclonal Ab) for flow cytometry and immunohistochemistry was purchased from Alexis Biochemicals (San Diego, CA). The anti‐CEACAM1 Ab (D1P4T, rabbit monoclonal Ab) for western blotting or immunoprecipitation was purchased from Cell Signaling Technology (Beverly, MA). The antimast cell tryptase Ab (AA1, mouse monoclonal Ab) and the antiglyceraldehyde 3‐phosphate dehydrogenase (GAPDH) Ab (6C5, mouse monoclonal Ab) were purchased from Abcam (Cambridge, MA), and the anticalcitonin Ab (rabbit polyclonal Ab) was from DAKO Cytomation (Glostrup, Denmark). The antiphospho‐Src (Tyr 416) Ab (rabbit polyclonal Ab), anti‐nonphospho‐Src Ab (7G9, mouse monoclonal Ab), antiphospho‐SHP‐1 (Tyr 564) Ab (D11G5, rabbit monoclonal Ab), and anti‐SHP‐1 Ab (C14H6, rabbit monoclonal Ab) were obtained from Cell Signaling Technology. Isotype control Abs were obtained from BD Biosciences (Bedford, MA). The secondary Abs were peroxidase‐labeled anti‐rabbit or anti‐mouse IgG antibodies (Santa Cruz Biotechnology, Santa Cruz, CA).

CEACAM1‐targeting shRNA lentiviral particles and the control particles (off‐target) were purchased from Santa Cruz Biotechnology. The establishment of CEACAM1‐knockdown or mock knockdown cells (off‐target) using LAD2, HMC1.1, HMC1.2 or TT as the target cells was performed according to the manufacturer's instructions.

The specific SFKs inhibitor PP1 and the specific SHP‐1 inhibitor PTP Inhibitor I, were purchased from Santa Cruz Biotechnology. These inhibitors were resolved in dimethyl sulfoxide (DMSO). We used PP1 at a concentration of 200 nmol/L and PTP Inhibitor I at a concentration of 50 *μ*mol/L in each experiment.

### PCR

A total of 5 x 10^6^ HuMCs and LAD2 cells were collected by centrifugation and processed with TRIzol (Invitrogen Life Technologies, Carlsbad, CA), after overnight cytokine‐depletion. The HMC1.1, HMC1.2, TT, K562, Jurkat, 293T, and HT‐29 cells were similarly processed but, as these cells were cytokine independent, no further overnight starvation was performed. The mRNA was extracted using RNeasy Plus kit according to the manufacturer's instructions (QIAGEN, Hilden, Germany).

One *μ*g of each sample was used in the reverse transcription (SuperScript III One‐Step RT‐PCR System; Invitrogen Life Technologies). The reverse transcription‐PCR primers for CEACAM1 were designed according to ref. 31 and ref. 32; FP49 (5’‐GCAACAGGACCACAGTCAAGACGA‐3’), BP59 (5’‐TGGAGTGGTCCTGAGCTGCCG‐3’), “exon6” (5’‐GGTTGCTCTGATAGCAGTAG‐3’), and “3’ untranslated region” (5’‐AGCCTGGAGATGCCTATTAG‐3’). FP49 and BP59 primers were used to distinguish 3L and 4L, and “exon 6” and “3’ untranslated region” primers were used to distinguish 3L/4L and 3S/4S. Complementary DNA synthesis and PCR amplification were performed with a DNA Engine PTC‐200 cycler (Bio‐Rad Laboratories) programmed with the following cycles: cDNA synthesis; 30 min at 55°C; denaturation: 2 min at 94°C; PCR amplification (30 cycles): 30 sec at 94°C (denature), 1 min at 60°C (anneal), 1 min at 72°C (extend); final extension at 10 min at 72°C.

For the real‐time PCR, one *μ*g of each sample was used in the reverse transcription (PrimeScript RT Master Mix; Takara, Ohtsu, Japan). The reverse transcription‐PCR primers for CEACAM1‐L and *β*‐actin were designed according to ref. [33]; CEACAM1‐L‐F (5’‐ACCCTGTCAAGAGGGAGGAT‐3’), CEACAM1‐L‐R (5’‐TGAGGGTTTGTGCTCTGTGA ‐3’), *β*‐actin‐F (5’‐TTGCCGACAGGATGCAGA ‐3’), and *β*‐actin‐R (5’‐GCCGATCCACACGGAGTACT‐3’). PCR amplification were performed with a Thermal Cycler Dice Real Time System II (Takara) using SYBR Premix Ex Taq, and programmed with the following cycles: an initial denaturation; 95°C for 30 sec; PCR amplification (40 cycles), 5 sec at 95°C (denature), 10 sec at 60°C (anneal); 15 sec at 72°C (extend); followed by a subsequent standard dissociation protocol.

### Western blotting

Cell lysates were prepared, and the proteins were separated by electrophoresis and probed for immuno‐reactive proteins as described 34.

### Flow cytometry

HuMCs which were cytokine‐depleted overnight and HMC1.2 cells were washed, fixed, and treated with RNase A. The cells were incubated with anti‐human CEACAM1 (GM8G5) or control Abs overnight at 4°C followed by anti‐mouse IgG‐FITC for 6 h at 4°C. The cells were then analyzed using a FACScan flow cytometer.

### Immunostaining

Immunohistochemistry was executed as before 24. After deparaffinization with xylene, tissue sections were rehydrated and pretreated with 0.3% hydrogen peroxide for 5 min. After steam heat for 40 min, anti‐human CEACAM1 (GM8G5), antimast cell tryptase or anticalcitonin Abs were added overnight at 4°C, following blocking background staining using Protein Block (X0909, DAKO Cytomation). Staining was performed using the ENVISION kit (DAKO Cytomation) according to the manufacturer's instructions. The successful CEACAM1 staining was confirmed by the positive staining of lymphocytes, which expressed CEACAM1 35. Mast cell tryptase‐positive cells were judged as neoplastic or nonneoplastic mast cells, and calcitonin‐positive cells were as medullary thyroid cancer cells or nonneoplastic C cells.

For immunocytochemistry, CEACAM1‐knockdown or mock TT cells were recultured in the 8‐well chamber slide glass, and fixed in 4% (v/v) paraformaldehyde. Anti‐CEACAM1 Ab (GM8G5) or isotype control Ab were added and incubation continued for 2 h at room temperature. Staining was performed using the ENVISION kit.

### Cell growth assay

The Cell Counting Kit‐8 (CCK‐8; Dojindo, Kumamoto, Japan) was used to evaluate the proliferation of the CEACAM1‐knockdown or mock LAD2, HMC1.1, HMC1.2, and TT cells. The CEACAM1‐knockdown or mock LAD2 cells were cultured overnight in cytokine‐free StemPro34. The CEACAM1‐knockdown or mock LAD2 cells were then recultured for 22 or 46 h at a density of 2–4 × 10^4^ cells/100 *μ*L of StemPro34 with 100 ng/mL rhSCF in the 96‐well dish (U‐bottom) again. The CEACAM1‐knockdown or mock HMC1.1 and HMC1.2 cells were cultured overnight in RPMI1640 + 10% FCS. The CEACAM1‐knockdown or mock HMC1.1 and HMC1.2 cells were then recultured for 22 or 46 h at a density of 3–4 × 10^4^ cells/100 *μ*L of RPMI‐1640 medium + 10% FCS in the 96‐well dish (U‐bottom) again. The TT cells were cultured overnight at a density of 5 × 10^4^ cells/100 *μ*L in F12 + 10% FCS in the 96‐well dish (Flat bottom), and recultured for 46 or 70 h after changing the new medium. In each assay, we added 10 *μ*L of CCK‐8 solution for the last 2 h and estimated the absorbance of the cultures at 450 nm, according to the manufacturer's instructions.

In other experiments, the HMC1.2 cells were cultured overnight in RPMI1640 + 10% FCS, and recultured for 22 h at a density of 4 × 10^4^ cells/100 *μ*L of RPMI1640 + 10% FCS with DMSO, PP1, or PTP Inhibitor I in the 96‐well dish (U‐bottom) again. The TT cells were cultured overnight at a density of 5 × 10^4^ cells/100 *μ*L in F12 + 10% FCS in the 96‐well dish (Flat bottom), and recultured for 46 h after changing the new medium with DMSO, PP1, or PTP Inhibitor I. Then, we added 10 *μ*L of CCK‐8 solution for the last 2 h and estimated the absorbance of the cultures at 450 nm.

### ELISA assay for the phosphorylation status of SHP‐1 and SFKs

1 × 10^6^ CEACAM1‐knockdown or mock HMC1.2 and TT cells were cultured overnight in the 96 well U‐bottom and Flat bottom plates, respectively. The cells were collected, and resolved in 200 *μ*L of assay buffer. One hundred *μ*L of the solution was used for ELISA assay for SHP‐1 (Assay Biotech, Sunnyvale, CA,), and another 100 *μ*L was used for ELISA assay for SFKs (Cell signaling Technology) according to the manufacture's protocol.

### Immunoprecipitation

Immunoprecipitation analysis was performed as described previously 20. We utilized the beads‐conjugated anti‐CEACAM1 Ab (D1P4T, rabbit monoclonal Ab) for this assay.

### Statistical analysis

Data were expressed as the means ± SE. Differences between groups were examined for statistical significance using Student's *t*‐test (Excel: Microsoft, Seattle, WA). A *P* < 0.05 indicated statistical significance.

## Results

### Expression of CEACAM1 in neoplastic and nonneoplastic human mast cells

**Figure 1:**
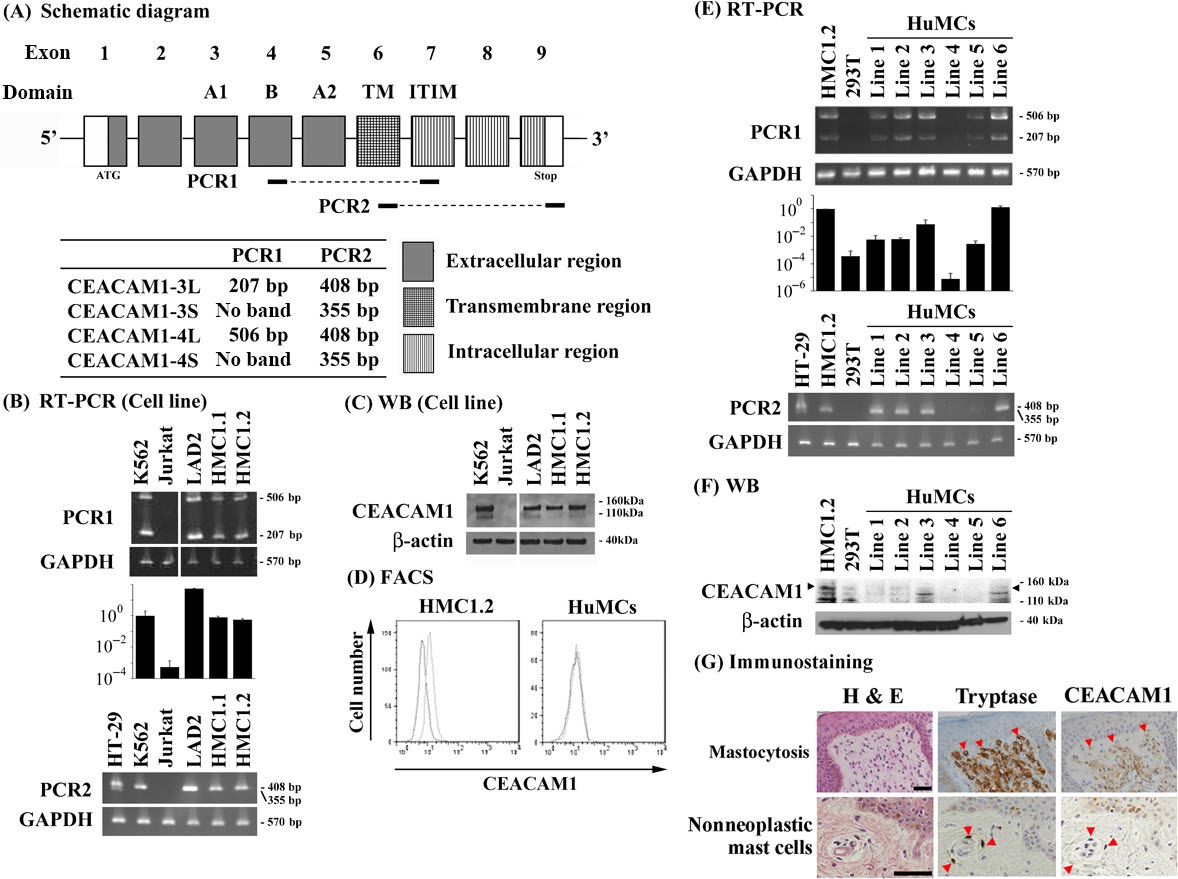
Human neoplastic mast cells express CEACAM1. (A) Primer design for reverse transcription PCR. (B) Reverse transcription PCR (representative bands) and real‐time PCR (relative copy numbers). (C) Western blotting for the neoplastic mast cell lines. The K562 cells were used as a positive control and the Jurkat cells as a negative control. (D) Flow cytometry for HMC1.2 and HuMCs. (E) Reverse transcription PCR (representative bands) and real‐time PCR (relative copy numbers). (F) Western blotting for HuMCs. Arrowheads indicate the expected size of CEACAM1 protein. (G) Immunostaining of skin mastocytosis cells derived from a patient with mastocytosis or chronic dermatitis, nonspecific. Both mastocytosis cells and nonneoplastic mast cells were shown as tryptase‐positive cells (red arrowheads), and the corresponding cells are also indicated by the red arrowheads in the right column. Bars: 50 *μ*m.

Initially, we examined the expression of CEACAM1 mRNA and protein in the LAD2, HMC1.1, and HMC1.2 neoplastic mast cell lines. Two sets of reverse transcription‐PCR primers for CEACAM1 mRNA were designed to distinguish between the 3L/4L and 3S/4S isoforms (Fig. 1A) 31, 32. K562 cells were used as a positive control for CEACAM1‐3L/4L, and HT‐29 cells were used for CEACAM1‐ 3S/4S 36, 37. Jurkat cells were used as a negative control for CEACAM1‐3L/4L or 3S/4S 38. As expected, mRNA for CEACAM1 was found in the K562 and the HT‐29 cells, but not in the Jurkat cells (Fig. 1B). CEACAM1 mRNA was also detected in the LAD2, HMC1.1, and HMC1.2 human mast cell lines, with the sizes corresponding to the 3L/4L isoforms (Fig. 1B). Real‐time PCR confirmed that LAD2, HMC1.1, and HMC1.2 expressed CEACAM1‐L mRNA equal to or as much as the level of K562 (Fig. 1B). CEACAM1‐S mRNA could not be detected in LAD2, HMC1.1, and HMC1.2 cells by reverse transcription PCR (Fig. 1B). To confirm the presence of CEACAM1 protein, we next examined protein expression by immunoblot analysis. Two bands were detected in the lanes associated with the K562, LAD2, HMC1.1, and HMC1.2 cell lines (Fig. 1C). Two bands were observed in these cells lines, but not the Jurkat cell line, consistent with the appropriate molecular weights of CEACAM1. Flow cytometry analysis showed that HMC1.2 expressed the CEACAM1 on the cell surface (Fig. 1D). These studies confirm expression of CEACAM1 in neoplastic mast cell lines that primarily express the CEACAM1‐L cytoplasmic tail based upon RT‐PCR.

To further examine whether or not CEACAM1 was expressed in primary cultured human mast cells, HuMCs were developed from peripheral blood progenitors of multiple donors, following which mRNA levels for CEACAM1 were examined by reverse transcription‐PCR and real‐time PCR. In these studies, mRNA for CEACAM1 was detectably in HMC1.2 cells as a positive control and nondetectable in 293T cells as negative control with variable detection in the HuMC lines (Fig. 1E). The expression levels of CEACAM1 protein were also variable in these cells and where in general very low and less than that observed in the HMC1.2 cell line (band at the level of arrowheads in Fig. 1F). Although a band was detected in the 293T cells and in HuMC lines 4 and 5, this band was viewed as nonspecific as it migrated below the positive control associated with the HMC1.2 cell line and consistent with the mRNA studies. Even in the HuMC line which expressed CEACAM1 most strongly, the expression of CEACAM1 could not be detectable by flow cytometry and was thus not detectable on the cell surface (Fig. 1D). From these data we conclude that although the primary cultured human mast cells can variably express CEACAM1 mRNA and low levels of protein they do not express detectable levels of CEACAM1 protein on the cell surface.

We next evaluated neoplastic mast cells derived from the mastocytosis patients. CEACAM1 expression on mast cells in the skin biopsies of mastocytosis patients was evaluated by immunohistochemical staining (Table 1). Neoplastic mast cells (mastocytosis) were distinguished by their morphology and tryptase‐positivity. Five of 19 cases (26.3%) stained positive with anti‐CEACAM1 antibody and representative photos are shown in Figure 1G. In contrast, all nonneoplastic mast cells identified in the nonmastocytosis specimens (4 cases) were negative for CEACAM1 by this method (Fig. 1G).

### Expression of CEACAM1 in human medullary thyroid carcinoma cells

**Figure 2:**
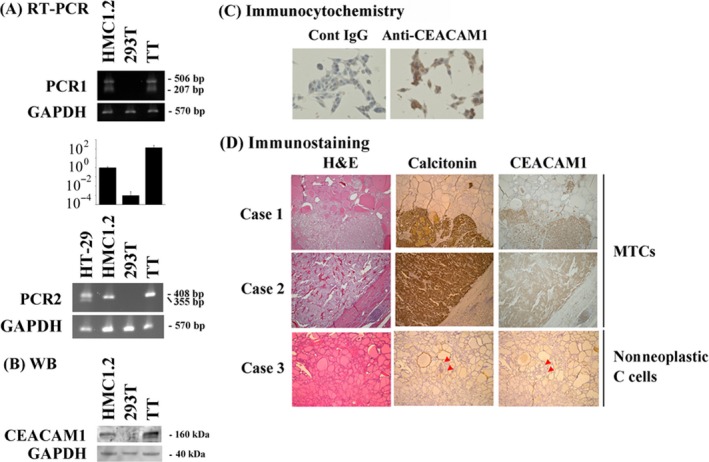
Human medullary thyroid carcinoma cells express CEACAM1. Human medullary thyroid carcinoma cell line TT expresses CEACAM1. (A) Reverse transcription PCR (representative bands) and real‐time PCR (relative copy numbers), (B) Western blotting, and (C) Immunocytochemistry were performed. The K562 cells were used as a positive control and the Jurkat cells as a negative control. (D) Immunostaining of clinical medullary thyroid carcinoma cells (Case 1 & 2) and nonneoplastic C cells (red arrowheads, Case 3). Bars: 50 *μ*m.

Subsequently, we explored other tumor cell lines harboring gain‐of‐function type mutations in tyrosine kinases, and found the expression of CEACAM1 mRNA and protein in a human medullary thyroid carcinoma (MTC) cell line, TT. As was the case for HMC1.2 cells, CEACAM1 mRNA for 3L/4L was also detected in the TT cells by reverse transcription‐PCR and real‐time PCR (Fig. 2A). We examined protein expression by immunoblot analysis and immunocytochemistry. These studies revealed that indeed CEACAM1 protein was expressed in TT cells (Fig. 2B and C).

We next evaluated human MTC cells using the pathological specimens resected surgically. CEACAM1 expression on human MTC cells in the pathological specimens was evaluated by immunohistochemical staining (Table 2). Sixteen of 22 cases (72.7%) stained positive with anti‐CEACAM1 Ab and representative cases are shown in Figure 2D. The nonneoplastic C cells, from which MTCs are thought to arise, were also positive for CEACAM1 from the patients with MTCs, though the C cells were CEACAM1‐negative in the patients with benign lesions, such as adenomatous goiter (Fig. 2D).

### CEACAM1 knockdown enhances the cell growth and the cell adhesion of LAD2, HMC1.1, and HMC1.2 cells, but suppresses the cell growth of TT cells

**Figure 3:**
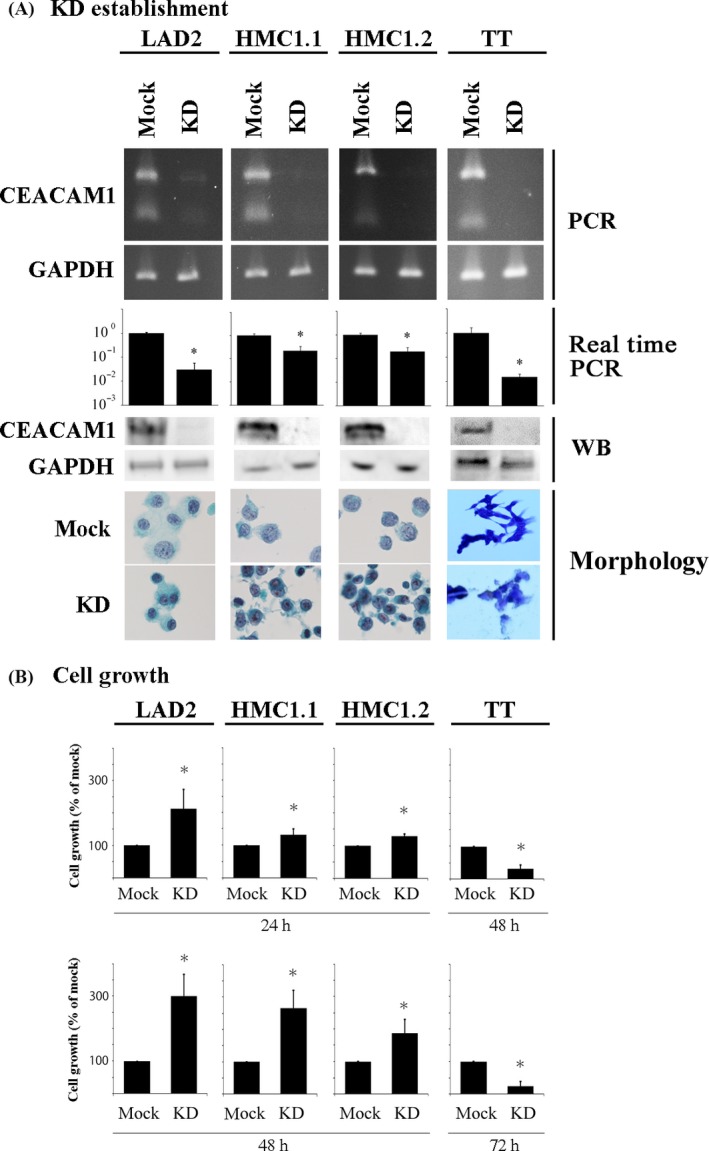
CEACAM1 knockdown enhances cell growth of LAD2, HMC1.1 and HMC1.2 cells, but suppresses that of TT cells. (A) Establishment of CEACAM1‐knockdown LAD2, HMC1.1, and HMC1.2 and TT cells. Reverse transcription PCR, real‐time PCR (relative copy numbers), Western blotting, and morphology (LAD2, HMC1.1 and HMC1.2 cells; Papanicolaou staining, TT cells; Diff‐Quik stain). (B) The CCK‐8 kit was used for the evaluation of the cell growth of CEACAM1‐knockdown or mock LAD2, HMC1.1, HMC1.2 and TT for the indicate time (*n* = 3, respectively). *P <* *0.05, when compared with the value of mock cells.

Following the determination that the human neoplastic mast lines LAD2, HMC1.1, and HMC1.2 and the MTC line TT exhibited significant expression of ITIM‐containing CEACAM1 isoforms, we next investigated the potential functional role for CEACAM1 in these cells. For this purpose, we utilized a knockdown method using shRNA and established CEACAM1‐knockdown LAD2, HMC1.1, HMC1.2 or TT cells (Fig. 3A).

We evaluated the effects of CEACAM1 knockdown on cell growth. We assessed the cell growth by the CCK‐8 assay. Growth of HMC1.2 cells, which is presumably KIT‐driven 17, 29, 30, was upregulated by the CEACAM1 knockdown when the cells were cultured in the U‐bottom wells for 24 or 48 h, though interestingly not in flat‐bottom wells, suggesting that cell‐cell contact was required for the inhibitory effect of CEACAM1 and its reversal on growth upon knockdown (Fig. 3B). Cell death is a contrary process to cell growth. The proportions of cell death were almost at equal levels between CEACAM‐knockdown and mock LAD2, HMC1.1, and HMC1.2 cells (data not shown). In contrast, growth of TT cells, which is presumably driven by mutated RET 25, was significantly downregulated by the CEACAM1‐knockdown in the either U‐bottom or flat‐bottom wells for 48 or 72 h (Fig. 3B). The proportion of cell death was not significantly different between mock and CEACAM1‐knockdown TT cells (data not shown).

### CEACAM1 knockdown downregulates the phosphorylation of SHP‐1 in HMC1.2 cells, and does that of SFKs in TT cells

**Figure 4:**
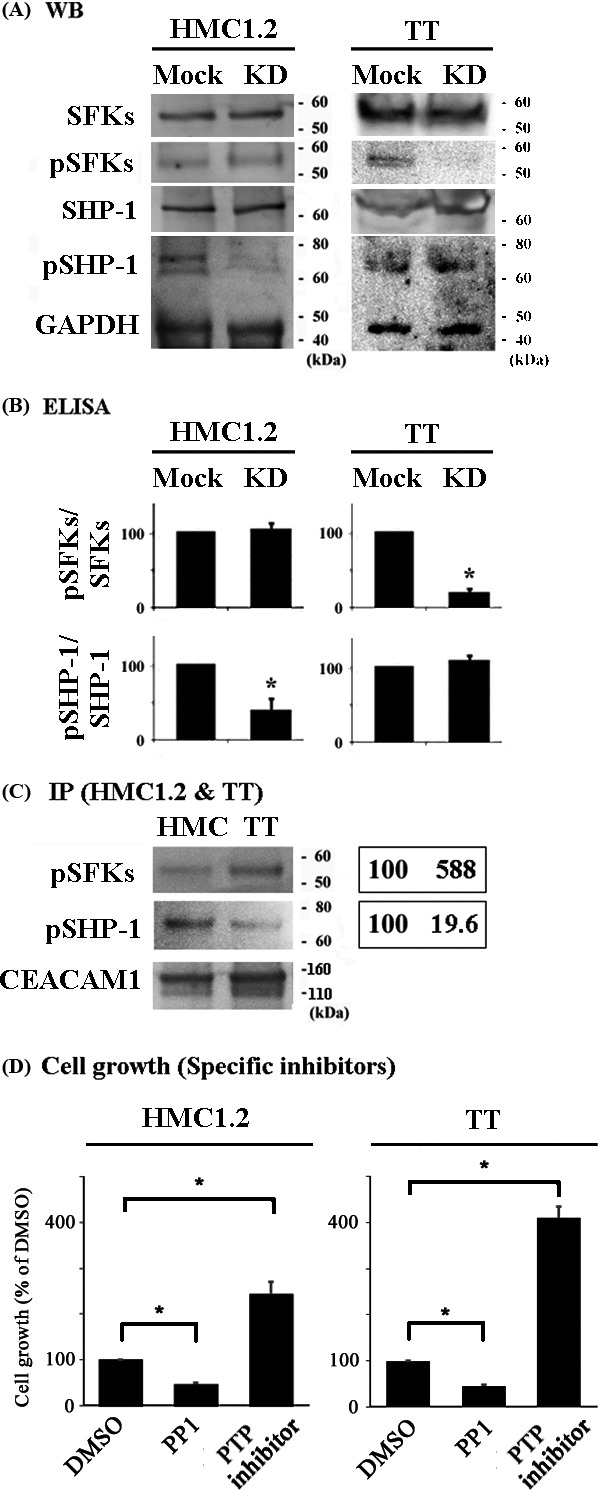
Activated SHP‐1 is preferentially associated with CEACAM1 in HMC1.2 cells, whereas activated SFKs are preferentially associated with CEACAM1 in TT cells. The phosphorylation status of SFKs and SHP‐1 in CEACAM1‐knockdown and mock HMC1.2 or TT cells. Western blotting. Data are representative of three individual experiments. (B) ELISA for phospho‐SHP‐1 or phospho‐SFKs in HMC1.2 or TT cells. Relative values when the values of mock cells are 100. *P <* *0.05, when compared with the value of mock cells. (C) The interaction of SFKs or SHP‐1 with CEACAM1 in HMC1.2 and TT cells. Left panel: Immunoprecipitation. Right panel: the average of relative values of the band intensities in the blots (*n* = 3), when the band intensities of HMC1.2 are normalized to 100. (D) The CCK‐8 kit was used for the evaluation of the effect of the specific inhibitor for SFKs PP1 and the specific inhibitor for SHP‐1 PTP inhibitor I on the cell growth of HMC1.2 (24 h) and TT (48 h) (*n* = 3, respectively). *P <* *0.05, when compared with the value of mock cells.

The inhibitory effect of CEACAM1 is mediated by the activation of SHP‐1 after its binding to the phosphorylated cytoplasmic tail of CEACAM1 resulting in the downregulation of critical tyrosine kinase‐mediated signaling events in lymphoid cells 1. SFKs on the other hand are typically activating and bind to multiple intracellular receptors including CEACAM1 which, in the case, results in CEACAM1 phosphorylation 9. We therefore examined the status of SHP‐1 and SFKs in relationship with CEACAM1 knockdown. We observed decreased SHP‐1 phosphorylation, suggesting decreased activation as a consequence of diminished CEACAM1 availability, but unchanged SFK phosphorylation upon CEACAM1‐knockdown in HMC1.2 cells in comparison to that observed in mock HMC1.2 cells (Fig. 4A and B). This suggests that in the absence of CEACAM1, there is unopposed activation of SFK in HMC1.2 cells.

In contrast, upon knockdown of CEACAM1 in TT cells, SHP‐1 phosphorylation was similar to that observed in mock TT cells and SFK phosphorylation was decreased (Fig. 4A and B). We could not detect any difference in the quantity of total SFKs and SHP‐1 (Fig. 4A), and in the status of SHP‐2 when comparing CEACAM1‐knockdown or mock HMC1.2 or TT cells (data not shown). This suggests that in TT cells, CEACAM1 is primarily engaged by SFK rather than SHP‐1.

Immunoprecipitaion with anti‐CEACAM1 Ab (D1P4T) showed that CEACAM1 interacted with SHP‐1 and SFKs in both HMC1.2 and TT cells (Fig. 4C). However, the quantity of phospho‐SHP‐1 which was observed to interact with CEACAM1 was higher than the amount of SFKs which interacted with CEACAM1 in HMC1.2 cells, with opposite findings observed in TT cells (Fig. 4C).

We assessed the growth of HMC1.2 and TT cells by adding the specific inhibitors for SFKs and SHP‐1. The administration with the specific inhibitor for SFKs PP1 decreased the cell growth of both cells, whereas that with the specific inhibitor for SHP‐1 PTP inhibitor I increased the cell growth of both cells (Fig. 4D). Thus, SFKs were confirmed to regulate cell growth of HMC1.2 and TT positively, and SHP‐1 was confirmed to regulate cell growth of the cells negatively.

## Discussion

CEACAM1‐L isoforms transduce inhibitory signals via ITIMs contained within the cytoplasmic tail which has previously suggested that CEACAM1 can function as a tumor suppressor 1. However, the expression of CEACAM1‐L is associated with a poor prognosis in many tumor types 26, 39, 40, 41, 42, 43. This discrepancy is sometimes explained by the involvement of CEACAM1‐L in angiogenesis, the ability of CEACAM1‐L to be associated with increased metastasis or tumor associated CEACAM1‐L ligation of inhibitory receptors on tumor infiltrating lymphocytes 4, 41, 42. In the latter case, CEACAM1 is expressed on activated T and NK cells, and regulates the function of these cells via homophilic binding to CEACAM1 or heterophilic binding to T‐cell immunoglobulin domain and mucin domain‐3 and NK gene complex group 2 member D (NKGD2), followed by the downregulation of antitumor immunity 4, 44. CEACAM1 also downregulates NKG2D ligand on the surface of tumor cells which potentially allows them to escape from the antitumor immunity induced by NKG2D‐expressing NK cells 45.

In this study, CEACAM1‐L expressed in a MTC cell line (TT) enhanced cell growth in association with preferential interactions with and activation of SFKs. This suggests that CEACAM1 association with and activation of SFKs may promote tumor cell growth as another explanation for the divergent roles ascribed to CEACAM1 in malignancy. In this regard, some inhibitors for SFKs have been reported to be effective for the treatment of MTCs 46, 47, and this study revealed an inhibitor for SFKs PP1 suppressed the growth of TT cells. These observations suggest their function might be via CEACAM1‐L‐associated or mutated RET‐associated SFK activation. On the other hand, TT cells exhibited a decreased interaction with phospho‐SHP‐1 when compared with HMC1.2 cells, suggesting that this decreased association may be related to the increased proliferation afforded by CEACAM1‐L expression in TT cells. Interestingly, there is a report that SHP‐1 suppresses the proliferation of TT cells when stimulated by somatostatin 48, and we here observed that an inhibitor for SHP‐1 PTP Inhibitor I enhanced the growth of TT cells. In addition, SHP‐1 has been reported to interact with mutated RET, but not to suppress mutated RET‐associated signals 49. It is therefore possible that sustained interaction of CEACAM1‐L with SFKs in TT, and potentially other MCTs, may force them into an activated stated that promotes uncontrolled proliferation.

In contrast, mastocytosis cells behaved differently. We detected variable levels of mRNA for CEACAM1 in normal mast cells derived from the blood of multiple donors, consistent with previous reports 50, 51. However, we observed very low levels of CEACAM1 protein in normal mast cells and could detect little evidence of CEACAM1 on the cell surface of them. In contrast, we observed significantly higher levels of CEACAM1 on neoplastic mast cells, based on the greater amounts of CEACAM1 protein detected in the HMC1.2 and LAD2 cell lines compared to that observed in the CD34^+^‐derived human mast cells from multiple donors. The expression of high levels of CEACAM1 expression in the neoplastic mast cells is consistent with our previous report of CEACAM1 expression on LAD3 cells, a sister cell line of the LAD2 cell line 13. Seemingly, the expression of CEACAM1‐L which can act as tumor suppressor in mastocytosis is contradictory. We are presuming that some signals constitutively activated in mastocytosis would induce the expression of CEACAM1‐L, and that the expression of CEACAM1‐L might explain the favorable prognosis of mastocytosis 14. The reason for the low expression of CEACAM1 protein in the nonneoplastic mast cells relative to the neoplastic mast cells despite detectable mRNA expression is currently unclear. Nevertheless, in T cells there is certainly a precedent for such a disconnection between mRNA and protein levels. Human peripheral blood T cells stimulated with IL‐2 plus phytohemagglutinin for 1 day express significant levels of CEACAM1 mRNA, but detectable levels of CEACAM1 protein was not observed 52. The difference in expression of CEACAM1 protein in the nonneoplastic versus neoplastic mast cells may thus reflect differences in mRNA handling or posttranslational processing of the protein, for example, through ubiquitination and proteosomal targeting rather than differences in the transcriptional regulation. As is the case for mastocytosis, CEACAM1 protein expression was observed in most MTCs and C cells with RET mutations, but not in C cells with normal RET. These results might suggest that similar mechanisms of posttranscriptional or ‐translational processing of CEACAM1 also exist in MTCs, although we did not study the CEACAM1 mRNA expression levels in the MTCs relative to C cells (counterparts as nonneoplastic MTCs) in the pathological specimens.

Gain‐of‐function mutations in KIT have been linked to myeloproliferative disorders, for example, acute myeloid leukemia (AML) and neoplastic mast cell (mastocytosis). In the case of mastocytosis, the D816V mutation in the catalytic domain of KIT is considered a major predisposing factor in the progression of disease, and approaches to downregulate KIT activation could be considered a reasonable strategy for the treatment of mastocytosis. A potential alternative approach to inhibiting KIT activity would be to engage specific ITIM‐containing inhibitory receptors expressed on the mast cell and AML surface with the potential of reversing responses downstream of activated KIT. Indeed, a number of mast cell surface inhibitory receptors have been demonstrated to function in this capacity 18, 19, 20, 21, 22, 23, 24. Our studies reported herein suggest that CEACAM1‐L, by preferentially being expressed on neoplastic mast cells and interacting with SHP‐1, may allow selective targeting of these cells providing applicability of this approach to the treatment of mastocytosis. This hypothesis seemed to be assisted by the observation that the administration with specific inhibitors for SHP‐1 PTP Inhibitor I enhanced the growth of HMC1.2 cells. There was no difference of AKT phosphorylation status in between CEACAM1‐KD and mock HMC1.2 (data not shown), therefore AKT inhibition would be necessary to treat mastocytosis completely in addition to CEACAM1‐related SHP‐1 activation. We previously reported that CD72 inhibited the growth of HMC1.2 via SHP‐1 activation, and AKT phosphorylation status was stable even after CD72 stimulation‐induced SHP‐1 activation 20.

The CEACAM1‐SFKs complex and the CEACAM1‐SHP‐1 complex were expected to transduce competitive signals in both mutated KIT‐harboring and mutated RET‐harboring cells. Both mutated KIT and mutated RET interact with and regulate the phoshorylation of SFKs 8, 46, 47, and are expected to induce the formation of the CEACAM1‐SFKs complex in the cells harboring mutated KIT or mutated RET. SHP‐1 also interacts with and is phosphorylated by both mutated KIT and mutated RET 48, 49, 53. Mutated KIT‐interacted SHP‐1 negatively regulates the signals and induce the degradation and regeneration of KIT in the cells harboring mutated KIT like HMC1.2 53, but mutated RET‐interacted SHP‐1 does not in the cells harboring mutated RET like TT 48, 49. We are thinking that the difference of SHP‐1 status in between mutated KIT‐harboring (mastocytosis) and mutated RET‐harboring (MTC) cells would result in the different level of the formation of the CEACAM1‐SHP‐1 complex, and the followed different ratio of the CEACAM1‐SFKs complex/ CEACAM1‐SHP‐1 complex.

## Conflict of Interest

Dr. Blumberg has a conflict of interest as being a consultant to Syntalogic Pharmaceuticals, Inc. which is developing therapies to inhibit CEACAM1. The other authors have no conflict of interest.
